# Exfoliation and Raman Spectroscopic Fingerprint of Few-Layer NiPS_3_ Van der Waals Crystals

**DOI:** 10.1038/srep20904

**Published:** 2016-02-15

**Authors:** Cheng-Tai Kuo, Michael Neumann, Karuppannan Balamurugan, Hyun Ju Park, Soonmin Kang, Hung Wei Shiu, Jin Hyoun Kang, Byung Hee Hong, Moonsup Han, Tae Won Noh, Je-Geun Park

**Affiliations:** 1 Center for Correlated Electron Systems, Institute for Basic Science (IBS), Seoul 151-742, Republic of Korea; 2 Department of Physics and Astronomy, Seoul National University (SNU), Seoul 151-742, Republic of Korea; 3 National Synchrotron Radiation Research Center (NSRRC), Hsinchu 30076, Taiwan; 4 Department of Chemistry, Seoul National University (SNU), Seoul 151-742, Republic of Korea; 5 Department of Physics, University of Seoul, Seoul 130-743, Republic of Korea

## Abstract

The range of mechanically cleavable Van der Waals crystals covers materials with diverse physical and chemical properties. However, very few of these materials exhibit magnetism or magnetic order, and thus the provision of cleavable magnetic compounds would supply invaluable building blocks for the design of heterostructures assembled from Van der Waals crystals. Here we report the first successful isolation of monolayer and few-layer samples of the compound nickel phosphorus trisulfide (NiPS_3_) by mechanical exfoliation. This material belongs to the class of transition metal phosphorus trisulfides (MPS_3_), several of which exhibit antiferromagnetic order at low temperature, and which have not been reported in the form of ultrathin sheets so far. We establish layer numbers by optical bright field microscopy and atomic force microscopy, and perform a detailed Raman spectroscopic characterization of bilayer and thicker NiPS_3_ flakes. Raman spectral features are strong functions of excitation wavelength and sample thickness, highlighting the important role of interlayer coupling. Furthermore, our observations provide a spectral fingerprint for distinct layer numbers, allowing us to establish a sensitive and convenient means for layer number determination.

Mechanical exfoliation of Van der Waals-stacked graphite provided a means to isolate monolayer and few-layer graphene sheets, and demonstrated that the electrical, optical, mechanical and chemical properties of two-dimensional (2D) materials vary strongly with the number of atomic layers^1^,^2^. For example, whereas graphite is a robust semimetal, monolayer graphene is a zero-bandgap Dirac metal, the electrical properties of which are strongly modulated by the influence of external factors such as adsorbates^3^ or the presence of a substrate^4^,^5^. The same exfoliation method has since been applied to a large number of layered Van der Waals materials, including insulating hexagonal boron nitride^5^, the high-temperature superconductor Bi_2_Sr_2_CaCu_2_O_8+*δ*_ (Bi-2212)^6^,^7^, and the family of stacked transition metal dichalcogenide materials, MX_2_, with tetravalent M^IV^ such as Nb, Mo, Ta, W, Re, and chalcogen X = S, Se, Te^6^,^8^. Amongst these, molybdenum disulfide (MoS_2_) has received particular attention due to its novel electronic properties, with a transition from an indirect bandgap found in bulk and multilayer MoS_2_ to a direct bandgap in monolayer MoS_2_^9^,^10^. Proceeding beyond their already fascinating properties, the isolated monolayers and multilayers of different stacked compounds can be reassembled to yield Van der Waals heterostructures and superlattices that may exhibit even more exotic behavior^11^, and this has fueled an ongoing search for new compounds with tailored physical and chemical properties that can be exfoliated as ultrathin sheets. In particular, for the design of spintronic devices^12^, Van der Waals materials that exhibit magnetic order would be highly desirable building blocks. However, with the exception of the antiferromagnetic parent compound of the high-*T*_*c*_ superconductor Bi-2212 mentioned above, the isolation of exfoliated monolayer or few-layer samples from Van der Waals materials with magnetic order at low temperature has not been reported. Thus, there is limited availability of materials both for the incorporation of spin order into complex Van der Waals heterostructures, and for fundamental studies of the impact of dimensionality onto magnetism in exfoliated samples with controlled numbers of layers.

The transition metal phosphorus trisulfide compounds, MPS_3_, form a separate family of stacked materials for which the interaction between layers occurs *via* Van der Waals forces^13^,^14^. All members comprise layers of covalently bonded (P_2_S_6_)^4−^ bipyramids and a honeycomb arrangement of divalent transition metal ions (Fig. 1a). The M^II^ site can be occupied by any 3d elements from V to Zn and by the 4d element Cd, and stoichiometric compounds have been synthesized with M = Mn, Fe, Co, Ni, Zn, Cd^15^. All of these compounds are stacked in an ABC fashion and have monoclinic crystal structure (space group 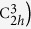
13,16; see Supplementary Table S1 for lattice parameters and key physical properties. Importantly, MPS_3_ exhibits magnetism for M = Mn, Fe, Co, Ni^15^,^17^,^18^, with a Curie-Weiss susceptibility at high temperature, and antiferromagnetic order at low temperature. The magnetic behavior is governed by competing direct M-M exchange and indirect M-S-M superexchange interactions within layers, as well as interlayer exchange interactions, and at low temperature, different antiferromagnetic ordering patterns are observed^15^,^17^,^18^. There are few theoretical predictions for the magnetic behavior of MPS_3_ materials in the ultrathin limit. Monolayer MnPS_3_ sheets have been predicted to exhibit strong coupling of the valley degree of freedom to the antiferromagnetic spin order on the Mn honeycomb lattice, allowing for strong valley polarization^19^. Nanosheets of MnPS_3_ have been calculated to form antiferromagnetic semiconductors that become ferromagnetic semimetals upon carrier doping, with opposite spin polarization for electron and hole doping^20^, and similar phenomena are thought to exist in other MPS_3_ compounds.

Another property of the MPS_3_ compounds that make the study of their exfoliated 2D variants attractive is the strong dependence of their optical properties on the M-site element, with absorption edge energies ranging between 1.5–3.5 eV^21^. Furthermore, due to their ability to accommodate extrinsic species in the Van der Waals gap, accompanied by dramatic modifications of their magnetic, optical and electrical properties, the MPS_3_ materials provide a suitable platform for intercalation-chemical studies^15^,^21^. In particular, the ability to reversibly intercalate lithium into the Van der Waals gap prompted great interest in the use of MPS_3_ as cathode materials for lithium batteries, with NiPS_3_ appearing to be the most promising candidate for potential application^22^.

Exfoliation of bulk CdPS_3_ and MnPS_3_ by an ion exchange method in aqueous solution resulted in a suspension of Cd_0.8_PS_3_ and Mn_0.8_PS_3_ monolayer flakes with lateral size 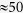 nm^23^,^24^. However, the experimental realization of stoichiometric ultrathin MPS_3_ sheets with lateral sizes in the micrometer range that allow for the study of intrinsic 2D material properties has not been reported so far.

## Results/Discussion

**Figure 1:**
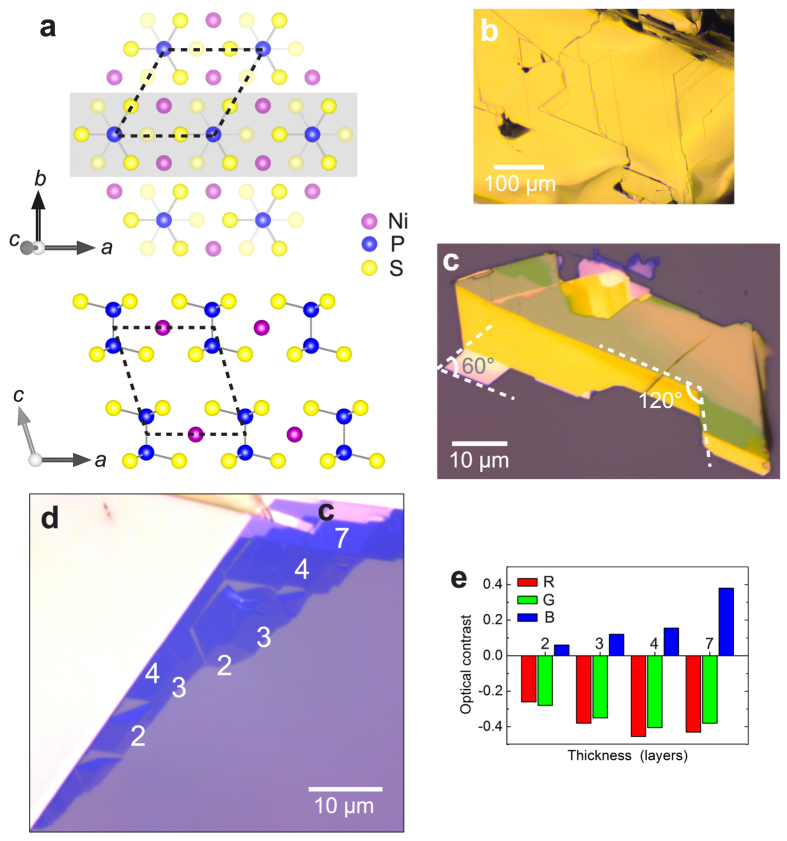
Atomic structure and optical characterization of exfoliated NiPS_3_. (**a**) Schematic crystal structure. View perpendicular to layers, only top layer shown (upper schematic). The atoms contained in the section shaded grey are shown in view parallel to layers (lower schematic). Unit cell (dashed outlines), covalent bonds within (P_2_S_6_)^4−^ anions (grey lines). (**b**) Brightfield microscope image of cleaved bulk NiPS_3_ samples. (**c**) Image of NiPS_3_ flake exfoliated onto oxidized silicon substrate, comprising mainly thick sheets (>10 layers). 60° and 120° angles indicated. (**d**) Image of thin exfoliated NiPS_3_ sheets, 2–7 layers indicated. (**e**) Optical intensity contrast of ultrathin NiPS_3_ sheets (2–7 layers), evaluated for red, green, and blue color channels separately, with reference to the substrate. (Photos in panels (**c**) and (d) were acquired at different illumination conditions to maximize visibility of relevant features).

In this article, we present the first experimental demonstration of MPS_3_ monolayer and multilayer samples isolated by mechanical exfoliation, for the case of NiPS_3_. Bulk samples of NiPS_3_ (Fig. 1b) are grown by a vapour transport method. Using the well-known Scotch tape method, we exfoliate NiPS_3_ flakes onto silicon substrates capped by 90 nm silicon oxide (SiO_2_). In the resulting NiPS_3_ sheets, we observe that sheet edges and boundaries between regions of different layer numbers frequently run in parallel, or are arranged at angles close to 60° and 120° (Fig. 1c). As in the case of exfoliated graphite, this observation can be attributed to preferential crack-propagation along the high-symmetry crystal axes during exfoliation.

Figure 1d shows an optical brightfield microscope image of a region containing thin NiPS_3_ sheets as well as thicker flakes. Thick flakes appear as opaque yellow areas, whereas atomically thin flakes exhibit discrete levels of optical contrast with respect to the substrate, allowing for unambiguous discrimination between regions of different layer number. For a semi-quantitative estimate, the optical image recorded on a CCD camera can be decomposed into its red, green, and blue intensity channels. For each color channel, the optical contrast between an *n*-layer (nL) flake and the substrate is calculated from the color intensities as 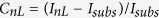
25. Fig. 1e demonstrates that discrete contrast levels are obtained for different sample thicknesses in each color channel.

**Figure 2:**
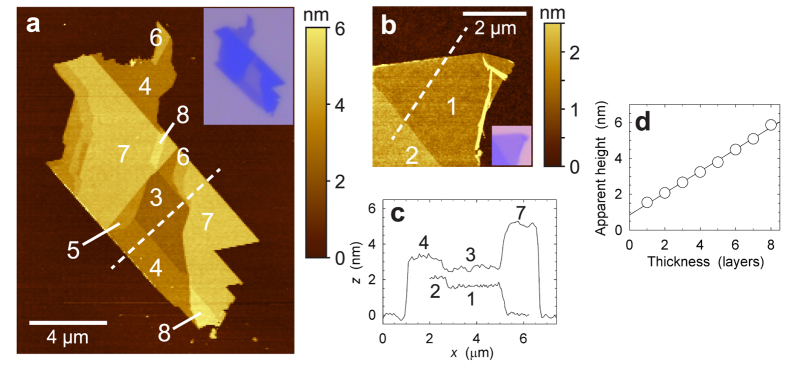
AFM characterization of exfoliated NiPS_3_. (**a**,**b**) Tapping-mode AFM topography image of ultrathin NiPS_3_ sheets, (**a**) 3–8 layers indicated, (**b**) 1 and 2 layers indicated. Insets: corresponding optical photographs. (**c**) Height profiles along the lines shown in (**a**,**b**). (**d**) Apparent layer heights evaluated from the AFM scans in (**a**,**b**), from 1 to 8 layers. Step heights between consecutive layers are ~0.6 nm, base height offset with respect to bare Si/SiO_2_ substrate is ~0.8 nm.

Layer numbers of thin NiPS_3_ sheets are assigned *via* a combination of optical contrast and height determination by atomic force microscopy (AFM). Figure 2a shows an AFM scan of a sheet comprising areas that are 3–8 layers thick, with lateral dimension 2–8 *μ*m, and Fig. 2b shows a NiPS_3_ sheet of monolayer and bilayer thickness, along with height profiles across the flakes and the SiO_2_ substrate (Fig. 2c). The measured layer heights shown in Fig. 2d illustrate that the spacing between NiPS_3_ flakes of different thickness is quantized in units of ~0.6 nm, consistent with the known bulk NiPS_3_ layer spacing 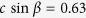 nm. However, the apparent height of monolayer NiPS_3_ with respect to the substrate, ~1.5 nm, is much greater than the interlayer spacing. We confirm that our assignment of monolayer and bilayer thicknesses is correct by performing an AFM scan on a sample containing bilayer and 5-layer sheets that overlap partially, allowing for an unambiguous calibration of our layer thickness scale (Supplementary Fig. S3). The phenomenon of an apparent base height offset is routinely observed in AFM measurements on exfoliated materials, such as graphene^1^,^26^, MoS_2_^27^,^28^,^29^, and other dichalcogenides^30^,^31^. It is commonly attributed to the presence of trapped adsorbates^32^ and to chemical contrast, *i.e.*, a difference in the interaction strength of the silicon AFM tip with the exfoliated flakes and the SiO_2_ substrate, respectively. Phase lag data that we acquire in AFM measurements (Supplementary Fig. S2) unambiguously show that significant chemical contrast between NiPS_3_ flakes and the SiO_2_ substrate is present, demonstrating that this mechanism contributes to the apparent height offset.

Raman spectroscopy is a powerful and popular characterization tool for bulk and exfoliated Van der Waals materials such as graphite/graphene and transition metal dichalcogenides^26^,^27^,^28^,^29^,^30^,^31^,^33^,^34^,^35^,^36^,^37^,^38^. The Raman spectra of bulk MPS_3_ and of (P_2_S_6_)^4−^ molecular anions in solution have been studied extensively both experimentally and theoretically^39^,^40^,^41^,^42^,^43^,^44^,^45^. Bulk NiPS_3_ is monoclinic (point group 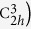, with a unit cell comprising two formula units (Ni_2_P_2_S_6_) in each layer. If the interlayer coupling is treated as a small correction, phonon modes can instead be labelled in terms of the D_3d_ point group of the hexagonal NiPS_3_ monolayer, and we follow this terminology to simplify comparison with the theoretical calculations of Bernasconi^44^. For spectra acquired in a backscattering geometry, first-order processes encompass phonons at the Brioullin zone center only. The irreducible representation of the zone center modes, 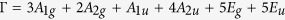, predicts 8 Raman-active phonon modes 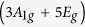, and all of these have been experimentally observed for bulk NiPS_3_^44^.

**Figure 3:**
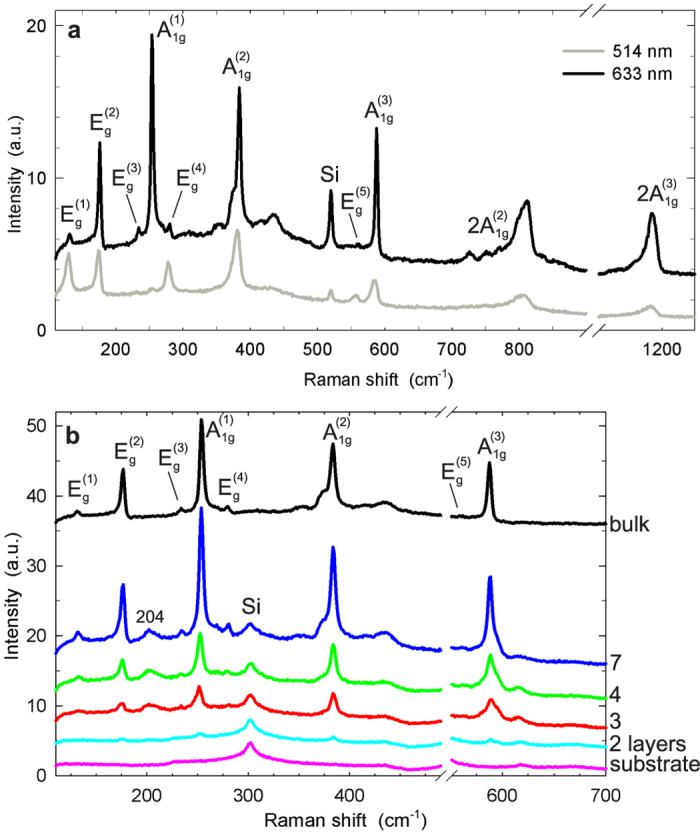
Raman spectra of exfoliated NiPS_3_. (**a**) Spectra of a thick sheet 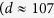 nm), acquired at excitation wavelengths 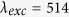 and 633 nm. These spectra are indistinguishable from those reported for bulk NiPS_3_. 5 in-plane 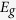 and 3 out-of-plane 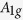 phonon modes are indicated. The Raman peak of silicon visible at 520 cm^−1^ stems from the substrate. (**b**) Spectra of thin NiPS_3_ sheets (2–7 layers), acquired at 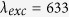 nm, together with the spectrum of a thick sheet shown in panel (**a**), and the substrate spectrum. All spectra were acquired in a single pass, under identical experimental conditions, on the sample shown in Fig. 1d. Spectra have not been scaled; data are offset vertically for clarity. The spectral region dominated by the first-order Raman peak of the silicon substrate around 520 cm^−1^ has been omitted. The spectral feature at ~300 cm^−1^ is a second-order Raman peak of silicon^56^.

In Fig. 3a, we show the Raman spectra of a thick NiPS_3_ flake (thickness ≈107 nm) that can be regarded as bulk material, measured at excitation wavelengths 514 and 633 nm. The spectra are consistent with previously reported Raman spectra of bulk NiPS_3_, and we assign the 8 expected Raman-active phonon modes according to that earlier work^44^. Since we probe the Raman response for two different excitation wavelengths, we can observe that the intensities of spectral features are a strong function of excitation energy. For excitation at 633 nm, all first-order modes are present, and the in-plane 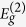 mode and all three out-of-plane 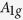 phonon modes dominate the spectrum. In contrast, in the spectrum collected at 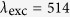 nm, the 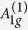 mode is very weak, and the 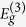 mode is not observed, whereas the 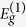 and 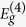 modes are enhanced; previous work^44^ reported an almost identical spectrum acquired at 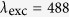 nm. This dependence on incident wavelength is presumably due to selective resonance enhancement controlled by electron-phonon coupling; however, to our knowledge no theoretical studies of this phenomenon that would allow for a more detailed discussion have been performed for MPS_3_ materials to date. Besides the spectral features assigned to first-order phonon processes, the Raman spectra contain several additional modes. For 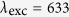 nm, the spectral region between 400–450 cm^−1^ contains broad peaks that were also observed in previous work^44^,^45^, and the spectral region between 700–850 cm^−1^ contains numerous peaks that have been interpreted as second-order processes^45^. Furthermore, we observe prominent features close to 1200 cm^−1^ that have not been previously reported and that we assign to the second overtone of the 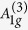 phonon mode.

Figure 3b shows the Raman spectra of thin NiPS_3_ sheets of 2, 3, 4, and 7 layers thickness, acquired for excitation 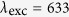 nm, along with the spectra of thick sheets and the substrate. All spectra were measured in a single pass on the NiPS_3_ sample shown in Fig. 1d, *i.e.*, sequentially and under identical experimental conditions. It is seen that as the layer number decreases, the spectral features undergo a significant evolution in several ways. Most visibly, while most Raman peaks are more intense in the 7-layer sheet than they are for bulk NiPS_3_, their intensity exhibits a marked progressive reduction in tetralayer, trilayer, and bilayer sheets. Intriguingly, the Raman features associated with the three 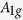 phonon modes also exhibit qualitative changes that make the spectra of bilayer through 7-layer NiPS_3_ sheets distinct and provide each of them with a unique fingerprint. Additionally, a peak that is not observed for bulk samples appears at 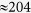 cm^-1^ for sheets of 3–7 layers thickness. For 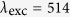 nm, the evolution of Raman spectra with sample thickness shown in Supplementary Fig. S6 is qualitatively similar but differs in a few important aspects. In particular, at this excitation wavelength, the spectrum of bilayer NiPS_3_ is almost indistinguishable from the SiO_2_ substrate, in contrast to the case of 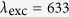 nm.

We note that photoluminescence is absent from all spectra acquired for 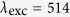 and 633 nm, for bilayer through 7-layer sheets and thick samples alike, up to the wavenumber limit of our spectra, 3000 cm^−1^. This observation rules out a transition of the indirect bandgap 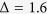 eV to a direct bandgap in thin NiPS_3_ sheets. Furthermore, we can place a lower bound on the direct bandgap of thin NiPS_3_, 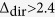 eV (corresponding to 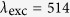 nm). We remark that it remains unknown whether a transition to a direct bandgap takes place in monolayer NiPS_3_, similar to the case of MoS_2_^9^,^10^. This is due to the technical challenge of obtaining monolayer sheets of sufficiently large size; the monolayer sheet depicted in Fig. 2b of ~3 *μ*m lateral size is suitably large, but it suffered degradation due to prolonged exposure to ambient atmosphere before Raman and photoluminescence spectra could be collected (see Supplementary Information). Other identified monolayer sheets had small lateral dimensions ~200 nm.

**Figure 4:**
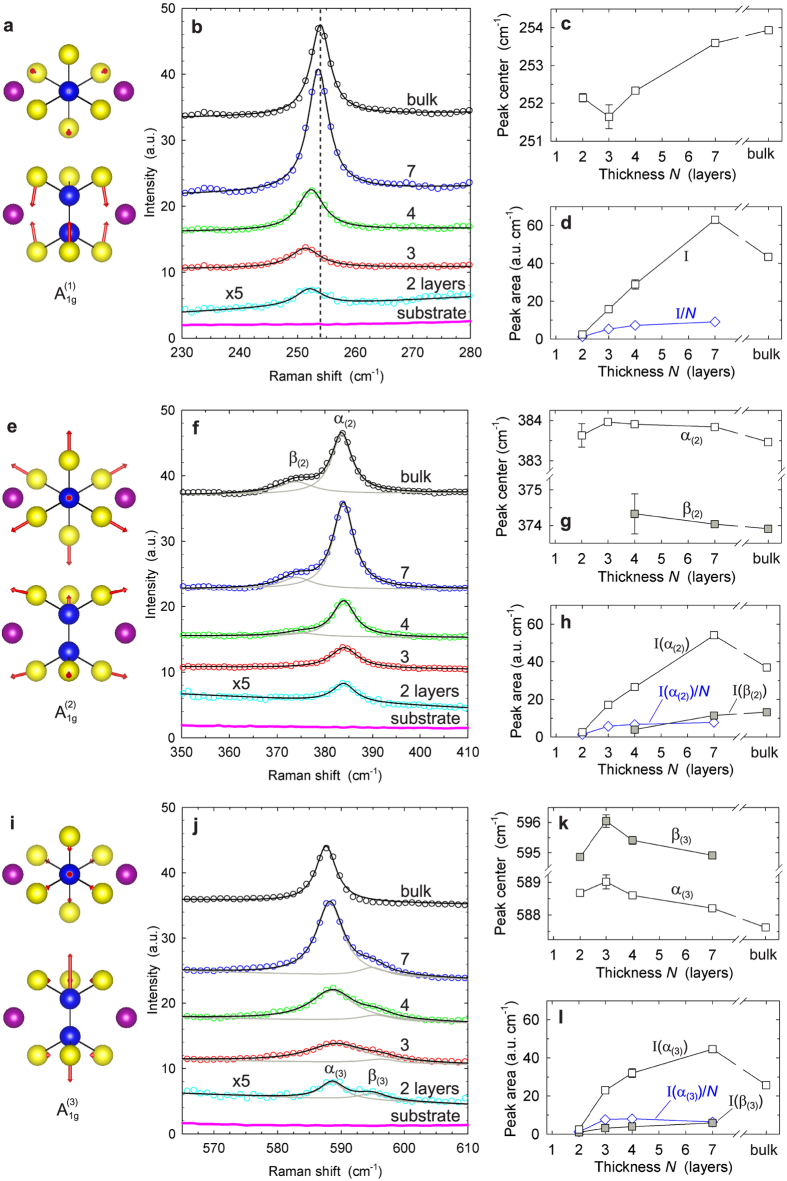
Out-of-plane *A*_1*g*_ phonon modes, and analysis of corresponding Raman spectral peaks of exfoliated NiPS_3_, for excitation at 633 nm. (**a**) Schematic representation (top view, side view) of vibrational amplitudes of Ni_2_P_2_S_6_ unit cell atoms in 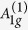 phonon mode^44^. (**b**) Detailed view of Raman data in corresponding spectral range, for thin sheets (2–7 layers), thick sheet, and silicon substrate. Spectra are overlayed with Lorentzian line shape fits. Data of bilayer sample have been magnified by factor 5. Spectra are offset vertically for clarity. (**c**) Central frequencies determined by Lorentzian peak fits in (b). Error bars correspond to spread of experimental data. (**d**) Integrated line shape intensities 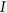 (=peak area) determined by Lorentzian peak fits in (**b**). (**e**–**h**) Analogous information for 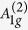 phonon mode. Raman spectra of 4-layer, 7-layer and thick NiPS_3_ sheets are composed of two distinct Lorentzian lines: individual Lorentzian fit curves (grey lines) and sum of fit curves (black lines). (**i**–**l**) Analogous information for 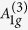 phonon mode. Raman spectra of thin sheets (2–7 layers) contain two superimposed Lorentzian lines.

In Fig. 4, we present a detailed analysis of the Raman spectral regions that contain the three out-of-plane 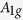 phonon modes, for 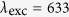 nm. The phonon modes can be visualized by indicating the associated vibrational amplitudes and directions of all atoms contained in the monolayer M_2_P_2_S_6_ unit cell, based on the calculations of Bernasconi^44^ (Fig. 4a,e,i). For all three 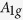 modes, the Raman line shapes can be adequately described by either a single Lorentzian curve or a superposition of two Lorentzian curves for thin sheets comprising 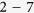 layers and for thick sheets (Fig. 4b,f,j), and we determine the positions and integrated intensities of these peaks by least-squares fits to spectral data. We discuss the trends observed in the extracted quantities in the following. Between 2 and 7 layers, the integrated intensity *I* scales approximately linearly with sheet thickness for all three 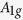 phonon modes, rising by a factor 20 ~ 25 over this range (Fig. 4d,h,l). Presumably, this increase is partly due to a combination of a volume effect and constructive interference enhancement. For bulk NiPS_3_, the intensity is significantly reduced with respect to 7 layers. Similar reductions of peak intensities with increasing sample thickness, from thin sheets to bulk, have been observed in the Raman response of other exfoliated materials such as graphene and MoS_2_. This effect is attributed to the suppression of constructive interference enhancement due to the absorption of incident and scattered light in the bulk sample^27^,^46^.

The intensity evolution for thin NiPS_3_ sheets contains an additional, more subtle anomaly. Intensity *I* and layer number *N* are not proportional to each other, and in particular, linear extrapolation to *N* = 0 would lead to large negative intensity offsets, suggesting that the Raman response of NiPS_3_ bilayers is anomalously weak. The evolution of the normalized intensity *I*/*N* with layer number (blue plots in Fig. 4d,h,l) highlights both the deviation from the expected proportionality and the very weak bilayer Raman emission. An explanation of this phenomenon in terms of damping caused by destructive interference is ruled out by the observation that all three 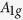 modes, despite their different emission wavelengths, exhibit similarly low responses. We speculate that instead this anomalously weak Raman response points to unusual intrinsic properties of bilayer NiPS_3_, or to a strong influence of coupling to the substrate. The low intensity of Raman spectra in NiPS_3_ bilayers is in stark contrast to materials such as graphene and MoS_2_, for which the Raman response intensity of mono- and bilayers is comparable to, or even exceeds that of bulk samples^27^,^46^.

We proceed to discussing our observations for the individual 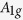 phonon modes. We focus on spectra collected at 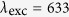 nm, while making reference to spectral data acquired at 514 nm excitation wavelength when these exhibit qualitative differences. The Raman peaks of the 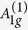 mode can be described well by a single Lorentzian line shape at 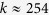 cm^−1^. The central frequency of this peak (Fig. 4c) undergoes a significant blue-shift as a function of sheet thickness, by 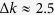 cm^−1^ between trilayer and bulk samples. For the 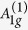 mode, atomic vibrations primarily involve rigid vertical motion of the sulfur planes, while the phosphorus and metal atoms remain at rest (Fig. 4a), *i.e.*, this mode strongly modulates the Van der Waals gap. Accordingly, the blue-shift 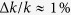 is most easily interpreted in terms of an 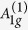 mode stiffening due to an increasing effective restoring force induced by the interaction with the added layers. Analogous observations have been reported for the out-of-plane phonon modes of exfoliated dichalcogenide materials, with similar magnitudes 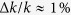
27,28,29,30,31,36,37,38. We remark that as in the case of bulk NiPS_3_, for 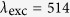 nm the 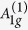 Raman mode is almost completely suppressed in sheets of 2−7 layers thickness (Supplementary Fig. S7).

The phonon modes 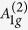 and 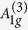 exhibit similarly dramatic changes with increasing sheet thickness, but their behavior is qualitatively different. Unlike the 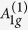 mode, the vertical components of their sulfur plane vibrations are weak (Fig. 4e,i), and no mode stiffening is observed with increasing layer number. Figure 4f shows Raman spectra of the 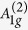 mode. For this mode, the response of bilayer and trilayer sheets is described well by a single Lorentzian peak at 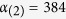 cm^−1^. In contrast, the spectra of 4− and 7-layer sheets and bulk NiPS_3_ flakes contain an additional component at 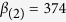 cm^−1^. For excitation at 514 nm, Raman spectra of the 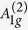 mode (Supplementary Fig. S7) evolve in a similar manner. The mechanism governing the appearance of the additional feature at 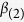 is unclear so far. We note that for MoS_2_, the appearance of a low-energy component in the 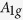 peak that increases in intensity with layer number has also been observed^47^. Generally, the breaking of translational crystal symmetry for thin sheets implies that they belong to symmetry classes that are different from the bulk material. This can lead to observable qualitative changes of the phonon modes in ultrathin sheets, as reported for the case of exfoliated dichalcogenide materials^48^. For NiPS_3_, the strong evolution of the feature at 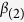, and its absence in bilayer and trilayer sheets, could be attributed to such differences in point group. Alternatively, the observed behavior could be assigned to the influence of the coupling to the SiO_2_ substrate, which is expected to affect very thin sheets most strongly.

Spectra of the 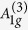 phonon mode shown in Fig. 4j vary in a similarly dramatic manner as a function of thickness, yet they show a converse trend. For thin sheets of 2−7 layers, two Lorentzian line shapes are present, at *α*_(3)_ = 588 cm^−1^ and 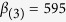 cm^−1^, whereas the spectrum of bulk NiPS_3_ contains only the dominant peak at 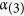. We will argue below that the mode at 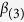 does contribute to second order Raman features, both for thin sheets and for thick samples. This implies that the phonon mode at 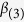 remains present in bulk NiPS_3_ but is Raman-inactive. The phenomena observed for excitation at 514 nm (Supplementary Fig. S7) are similar: for 4- and 7-layer sheets, two peaks can be resolved at 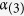 and 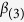, whereas in thick sheets the peak at 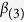 is absent. In addition, we observe a lower-energy peak at 583 cm^-1^ that is unique to bulk NiPS_3_. As before, the fact that thin sheets and bulk NiPS_3_ belong to different point groups might explain the invisibility of the mode at 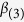 for bulk samples.

Among the five doubly degenerate in-plane phonon modes of NiPS_3_, the 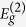 mode is most prominent. Supplementary Figure S8 shows the evolution of the corresponding spectral region with sample thickness, for 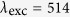 and 633 nm. In addition to the 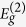 main peak centered at 176 cm^−1^, a low-energy shoulder located at 167 cm^−1^ can be resolved for 3–7 layers and thick sheets. Significantly, an additional peak that has not been observed in bulk NiPS_3_ appears at ~204 cm^−1^ in thin sheets, and for 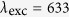 nm this peak can be clearly resolved for trilayer, tetralayer, and 7-layer sheets. The same consideration of translational symmetry breaking can be invoked to explain the appearance of this new spectral feature.

At this point, we briefly review the dependence of Raman-active phonon modes on the excitation wavelength. It was pointed out above that the 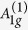 phonon mode is suppressed for excitation at 514 nm but dominates Raman spectra for 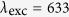 nm. The origin for this strong enhancement is unclear so far. In contrast, the remaining dominant modes 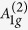, 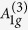, and 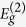 have a strong Raman response at both excitation wavelengths, and their intensity ratios 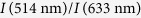 (Supplementary Fig. S9) exhibit a significant evolution with sheet thickness. With decreasing layer number, the intensity ratios exhibit a progressive enhancement over the bulk NiPS_3_ values for all three phonon modes. This observation could be attributed to a thickness-dependence of the electronic structure of few-layer sheets. For bilayer NiPS_3_, the observed trend is reversed abruptly as all intensity ratios vanish. This reversal reflects the absence of a Raman response in bilayers at 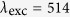 nm discussed previously, and it highlights that bilayer NiPS_3_ behaves qualitatively differently from sheets of greater thickness, including trilayers.

**Figure 5:**
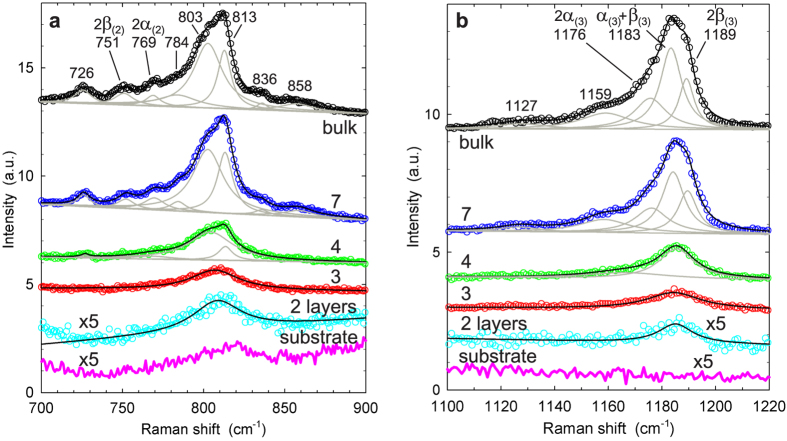
Raman spectral features of second order processes, at *λ*_*exc*_ = 633 nm. (**a**) Detailed view of spectral range containing 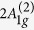 second-order processes. Spectra are described by superposition of up to 8 Lorentzian peak shapes: individual Lorentzian fit curves (grey lines) and sum of fit curves (black lines). Labels state wavelength shifts (in cm^−1^) of individual peak centers. Data of bilayer sample and substrate have been magnified by factor 5. Spectra are offset vertically for clarity. (**b**) Spectral range containing 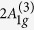 second-order processes. Spectra are described by up to 5 Lorentzian line shapes. See the text for details.

The Raman spectra of bulk NiPS_3_ and thin sheets contain several very distinctive features that arise from two-phonon processes. In the wavenumber region 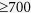 cm^−1^, the Raman response is dominated by two clusters of spectral peaks. Figure 5a shows the region between 700–900 cm^−1^, in which second-order processes have been observed but not analyzed in previous work^45^. This region contains numerous spectral features, and we perform a least-squares fit analysis to attain a more detailed understanding. For bulk and 7-layer NiPS_3_, spectra are adequately described by a superposition of 8 Lorentzian line shapes, centered at wavenumbers provided in the figure labels. We argue that at least a subset of these spectral peaks correspond to second-order overtones of the 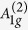 phonon mode: doubling the wavenumber shifts of the 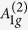 modes' two component peaks leads to 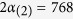 cm^−1^ and 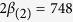 cm^−1^, in close agreement with the observed Lorentzian peaks centered at 769 and 751 cm^−1^, respectively. The physical mechanisms that lead to the appearance of the two dominant peaks at 803 and 813 cm^−1^ as well as the remaining smaller components are not clear at present. The intensities of all peaks decrease strongly as the layer number is reduced. For tetralayer NiPS_3_, four Lorentzian lines can be resolved in total; whereas the two dominant peaks remain clearly visible, the peaks at 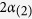 and 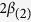 merge into a single feature that cannot be resolved further. For trilayer and bilayer sheets, a single peak described adequately by a Lorentzian line shape is visible at the position of the two previously dominant features. The evolution of the same spectral region for excitation at 514 nm is shown in Supplementary Fig. S10. For 7-layer sheets, spectra measured at the two excitation wavelengths are qualitatively similar, and four of the previously observed Lorentzian components are clearly resolved for 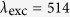 nm. In striking contrast, spectra of bulk NiPS_3_ differ greatly between the two excitation wavelengths; at 514 nm, only two strongly attenuated broad peaks can be resolved.

Figure 5b shows the Raman response in the range 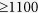 cm^−1^, which contains a cluster of spectral peaks that has not been previously reported. Using an analogous least-squares fit procedure, we decompose the spectrum for bulk and 7-layer NiPS_3_ into 5 Lorentzian lines. The three lines that are comprised in the dominant spectral feature can be attributed to second order overtones and combination modes of the 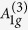 phonon mode: the three possible combinations of that mode's two components lead to 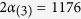 cm^−1^, 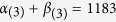 cm^−1^, and 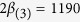 cm^−1^, in close agreement with the observed peak positions at 1176, 1183, and 1189 cm^−1^, respectively. There is no clear interpretation so far for the two additional peaks that are visible at lower energies. As before, all peak intensities decline rapidly with decreasing layer number. For tetralayer NiPS_3_, two Lorentzian lines are sufficient to describe the spectra, and for trilayer and bilayer sheets a single peak is resolved. Analogous to earlier comments, measurements of the 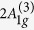 spectral region at 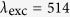 nm (Supplementary Fig. S10) resolve far fewer component peaks for bulk NiPS_3_ and 7-layer sheets, and in the bulk case the collected spectrum is greatly attenuated.

Our survey of the first- and second-order spectral features contained in the Raman response of NiPS_3_ sheets of 2, 3, 4, and 7 layer thickness, and of bulk NiPS_3_, demonstrates that for each sample thickness a qualitatively unique Raman spectrum is obtained. In particular, bilayer NiPS_3_ is identified by its characteristic dependence on incident wavelength: at 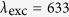 nm all dominant phonon modes are present, whereas at 514 nm the Raman spectrum is indistinguishable from the spectrum of the substrate. Trilayer and tetralayer NiPS_3_ are discriminated by the spectral positions of their 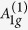 peaks, as well as by the qualitative differences between their 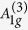 peak shapes (Fig. 4j and Supplementary Fig. S6). For thicker sheets, our observations suggest that the position of the 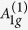 peak and the linear rise of the 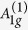 and 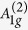 mode intensities allow for layer number identification at least up to 7-layer NiPS_3_. The existence of such spectral fingerprints can be utilized for a rapid and convenient determination of layer numbers in thin NiPS_3_ flakes.

## Conclusion

We have reported the first successful fabrication and characterization of ultrathin transition metal phosphorus trisulfide (MPS_3_) sheets, by micromechanical exfoliation of NiPS_3_, down to isolated monolayers. Further, we have demonstrated that the Raman spectra of thin NiPS_3_ sheets are drastically different from the bulk material, and vary strongly between sheets of different layer numbers. The key significance of our results is that bulk MPS_3_ compounds exhibit magnetism, and antiferromagnetic ordering strongly influenced by interlayer coupling is known to take place for M = Mn, Fe, Co, Ni at moderately low temperature. Magnetic order in bulk MPS_3_ crystals has previously been studied by means of Raman spectroscopy, which detects the modification of phonon modes due to a magnetic superstructure below the Néel temperature^41^,^42^,^43^, and this technique can be extended to exfoliated MPS_3_ sheets using a micro-Raman spectrometer for sufficiently large flakes (greater than the laser spot size ~1 *μ*m). If MPS_3_ sheets can be deposited onto a conducting substrate or covered with a conducting coating to prevent electrostatic charging, X-ray photoemission electron microscopy is a suitable tool to characterize antiferromagnetic order directly with high spatial resolution ~50 nm^49^,^50^. Thus, our work lays the foundation for the study of magnetic order and competing magnetic exchange interactions in thin MPS_3_ crystals of well-defined layer numbers, for an exploration of the impact of translational symmetry breaking and proximity of a substrate onto magnetic ordering phenomena, as well as for the application of spin-ordered monolayer and multilayer sheets as building blocks in Van der Waals heterostructures.

## Methods

NiPS_3_ crystals are grown by vapour transport method using 5% excess sulfur^51^. X-ray diffraction analysis (Rigaku Miniflex II) of flat crystallites shows that they grew in (001) orientation. Chemical composition analysis using a JEOL-EPMA provides a stoichiometry of Ni_0.959_P_1.000_S_2.976_, with a relative spread ±1%.

Bulk crystals are mechanically exfoliated onto oxidized silicon wafer pieces with adhesive tape (Ultron Systems, P/N 1007R), and adhesive residues are removed by immersion in trichloroethylene solvent^52^. To enable fast identification of exfoliated few-layer flakes by visual inspection, the thickness of the silicon oxide layer has to be chosen adequately to maximize optical interference contrast^53^. For the measured refractive index of bulk NiPS_3_ at ~550 nm, 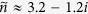, the optimum oxide thickness is 90 nm. Exfoliated samples are stored in a vacuum desiccator to minimize aging due to ambient exposure. If stored in ambient air, aging of exfoliated NiPS_3_ sheets results in a reduction of the optical contrast visible in optical microscope images after about one week, accompanied by a strong reduction of peak intensities in Raman spectra; AFM measurements show that aging leads to an increase in the apparent thickness of NiPS_3_ sheets by ~1 nm (see Supplementary Fig. S4). Heat treatment in ambient air results in a dramatic acceleration of sample aging. While these observations suggest that a progressive chemical degradation of exfoliated samples occurs, the detailed mechanism of this phenomenon warrants further study.

AFM characterization is performed with a Park XE-100 instrument, using dynamic force mode (cantilever spring constant ~40 N/m) in the repulsive force regime. AFM data are evaluated using the Gwyddion software package^54^. Micro-Raman measurements are performed in ambient on a Renishaw inVia instrument, using a 50x microscope objective. Spectra are collected for excitation wavelengths 514.5 and 632.8 nm, using gratings containing 2400 and 1800 grooves/mm, respectively. Measurements cover the wavenumber range 100–3000 cm^−1^. The silicon substrate’s Raman peak at 520 cm^−1^ is used as an internal calibration. At a laser spot size 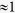 *μ*m, keeping the illumination power at ≤1 mW produces no observable sample damage or changes in the acquired spectra. Each spectrum is acquired with an integration time of 10 seconds. All spectra presented in this article have been collected in a single measurement session, such that experimental conditions are identical for all spectral data. Prior to each spectral acquisition, the focus of the laser was readjusted to coincide with the plane of the NiPS_3_ flake studied. All graphs showing Raman spectral intensities use identical units (1 a.u. = 1000 CCD counts). Phonon mode visualizations were drawn with VESTA^55^.

## Additional Information

**How to cite this article**: Kuo, C.-T. *et al.* Exfoliation and Raman Spectroscopic Fingerprint of Few-Layer NiPS_3_ Van der Waals Crystals. *Sci. Rep.* **6**, 20904; doi: 10.1038/srep20904 (2016).

## Supplementary Material

Supplementary Information
