# Supplementary material for: Exfoliation and Raman Spectroscopic Fingerprint of Few-Layer NiPS3 Van der Waals Crystals
Source: Sci Rep. 2016 Feb 15;6:20904. doi: 10.1038/srep20904 (PMC4753463; doi:10.1038/srep20904)
Supplement: Supplementary Information [file srep20904-s1.pdf]

Supplementary Information: Exfoliation and Raman Spectroscopic Fingerprint of Few-Layer $NiPS_3$ Van der Waals Crystals

Cheng-Tai Kuo[1,2,+], Michael Neumann[1,2,+], Karuppannan Balamurugan[1,2], Hyun Ju Park[1,2], Soonmin Kang[1,2], Hung Wei Shiu[3], Jin Hyoun Kang[4], Byung Hee Hong[4], Moonsup Han[5], Tae Won Noh[1,2,*], and Je-Geun Park[1,2,]**

[1]Center for Correlated Electron Systems, Institute for Basic Science (IBS), Seoul 151-742, Republic of Korea
[2]Department of Physics and Astronomy, Seoul National University (SNU), Seoul 151-742, Republic of Korea
[3]National Synchrotron Radiation Research Center (NSRRC), Hsinchu 30076, Taiwan
[4]Department of Chemistry, Seoul National University (SNU), Seoul 151-742, Republic of Korea
[5]Department of Physics, University of Seoul, Seoul 130-743, Republic of Korea
[+]these authors contributed equally to this work
[*]twnoh@snu.ac.kr
[**]jgpark10@snu.ac.kr

Physical properties of bulk MPS_3 Van der Waals crystals

The growth of stoichiometric MPS_3 crystals has been reported for M = Mn, Fe, Co, Ni, Zn, Cd.[1] The optical properties depend strongly on the choice of M-site element; absorption edge energies range between 1.5 - 3.5 eV.[2] Furthermore, for M = Mn, Fe, Co, Ni, bulk MPS_3 exhibits antiferromagnetic order at low temperature; the magnetic signature and properties of these compounds differ strongly for different M-site elements.[1,3,4] Table S1 lists the lattice parameters and key physical properties reported for all stoichiometric bulk MPS_3 compounds.

M^{II}-site atom	Mn	Fe	Co	Ni	Zn	Cd	
a (Å)	6.077	5.947	5.901	5.812	5.9717	6.218	refs. 5,6
b (Å)	10.254	10.300	10.222	10.070	10.3424	10.736	
c (Å)	6.796	6.722	6.658	6.632	6.7565	6.867	
β (°)	107.35	107.16	107.17	106.98	107.139	107.58	
Θ (K)	-217	+104	-116	-712	-	-	refs. 1,3
T_N (K)	82	116	122	155	-	-	refs. 1,3
absorption edge (eV)	3.0	1.5		1.6	3.4	3.5	ref. 2
R(300 K) (Ω cm)		2.5×10^4		10^9			ref. 2

Supplementary Table S1. Structural and physical properties of MPS_3 compounds, for M = Mn, Fe, Co, Ni, Zn, Cd. Monoclinic (space group C^3_{2h}) lattice parameters a, b, c, β, Curie-Weiss temperature Θ, antiferromagnetic ordering temperature T_N, and resistivity R at 300 K.

Bulk $NiPS_3$ sample growth and characterization

$NiPS_3$ crystals are grown by vapour transport reaction.[7] Inside an argon-filled glove box, elemental powders (purchased from Sigma-Aldrich) of nickel (99.99% purity), phosphorus (99.99%) and sulfur (99.98%) are weighed and mixed in stoichiometric ratio 1:1:3 and an additional 5 wt-% of sulfur, with a total mass $\sim$ 1 g. The mixture is ground into a fine powder, which is then sealed inside a fused quartz tube in vacuum ($\sim 10^{-3}$ mbar). The vapour transport growth method utilizes two regions at

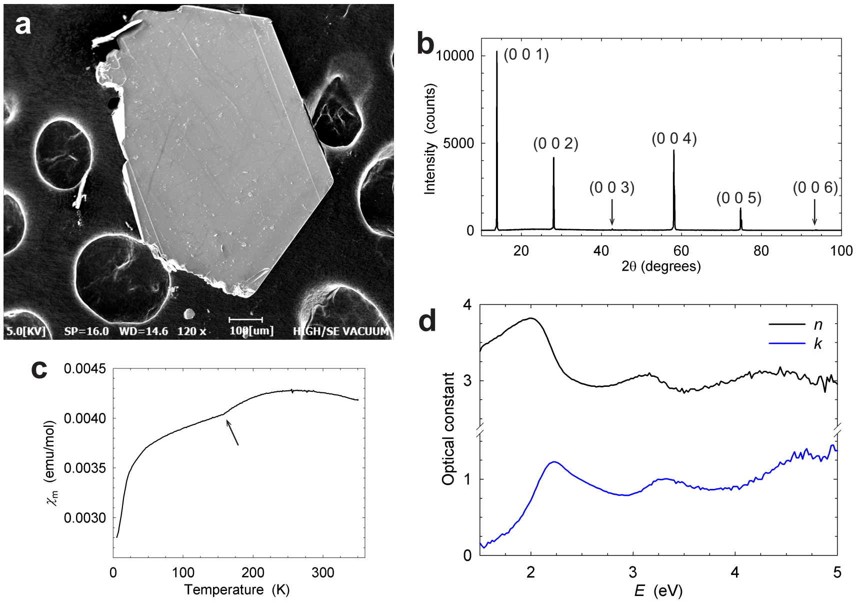

Supplementary Figure S1. Characterization of bulk $NiPS_3$. (a) Electron microscope image. (b) X-ray diffraction spectrum. (c) Molar magnetic susceptibility. (d) Energy dependence of real and imaginary terms of the complex index of refraction $\tilde{n} = n - \mathrm{i}k$, measured by ellipsometry.

different temperatures $T_1 > T_2$, referred to as charge and growth zones, respectively. We perform crystal growth in a two-zone tube furnace, at $T_1 = 720°$C and $T_2 = 690°$C, over the course of 10 days. The resulting flat crystallites are cleaned in ethanol.

Figure S1a shows the surface morphology of a typical as-grown $NiPS_3$ crystallite of ~ 0.5 mm $\times$ 0.8 mm lateral size imaged on a COXEM EM-30 scanning electron microscope. The $\theta - 2\theta$ X-ray diffraction spectrum of a $NiPS_3$ crystallite is measured on a Rigaku Miniflex II system, using the Cu K_α line. The spectrum (Fig. S1b) comprises $(00l)$ reflections for $l = 1-6$. The lattice spacing determined from peak positions is 6.35 ± 0.02 Å, consistent with the previously reported value $c \sin\beta = 6.34$ Å.[5] Figure S1c shows the temperature-dependent magnetic susceptibility of bulk $NiPS_3$, for the range $5-350$ K, measured on a $NiPS_3$ crystallite of mass 5.1 mg using a commercial magnetometer (MPMS5XL, Quantum Design) in a magnetic field 300 Oe. The sharp kink at 157 K marked by an arrow in suceptibility data identifies the magnetic ordering temperature T_N, in agreement with previous reports.[2,3,7,8] The refractive index n and extinction coefficient k of bulk $NiPS_3$ are determined as a function of wavelength (Fig. S1d) from spectroscopic ellipsometry measurements on a cleaved $NiPS_3$ crystallite, using a J.A. Woollam VASE instrument. At $E = 2.25$ eV (corresponding to $\lambda \simeq 550$ nm), the complex index of refraction is $\widetilde{n} \simeq 3.2 - 1.2i$. In order to maximize the visibility of exfoliated $NiPS_3$ on silicon substrates capped by thermal oxide, we use $\widetilde{n}(550$ nm) to compute the optical intensity contrast of $NiPS_3$ as a function of SiO_2 thickness. We calculate an maximum contrast $C \sim -0.3$ for 90 nm oxide thickness, consistent with the experimentally found contrast in the green color channel shown in Fig. 1e.

Apparent base height offset of $NiPS_3$ sheets in AFM measurements

In the main manuscript and Fig. 2, we show that the apparent height of monolayer $NiPS_3$ sheets measured by ac-mode AFM is ~ 1.5 nm, far greater than the interlayer spacing ~ 0.63 nm known from X-ray diffraction,[5] *i.e.*, we observe an apparent base height offset ~ 0.8 nm. This phenomenon is frequently observed in AFM measurements on exfoliated materials, such as graphene,[9,10] MoS_2,[11–13] and other dichalcogenides.[14,15] While in ac-mode AFM, offset artifacts of this kind can be induced by incorrectly operating the cantilever in a bistable regime where it switches between attractive and repulse interactions with the sample,[16,17] we choose a sufficiently low oscillation amplitude setpoint to operate in the repulsive regime only. Adsorbates trapped under the exfoliated sheet can be a second cause of base offsets that has been demonstrated for graphene deposited on SiO_2 and mica substrates;[18,19] we cannot prove or rule out this mechanism in the case of our $NiPS_3$ samples. However, we can demonstrate yet another mechanism commonly leading to base offsets, namely chemical contrast, *i.e.*, a difference in the interaction strength of the silicon AFM tip with the exfoliated flakes and the SiO_2 substrate, respectively. Figure S2 presents topography and cantilever phase lag images recorded simultaneously by AFM on the monolayer and bilayer $NiPS_3$ sample also shown in Fig. 2b of the main manuscript. The phase lag is a measure of the energy loss that the cantilever experiences

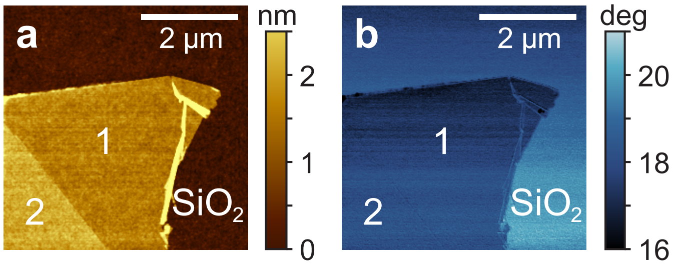

Supplementary Figure S2. Chemical contrast in ac-mode AFM scans. (a) Topography and (b) phase lag images of exfoliated $NiPS_3$ monolayer and bilayer sheets on SiO_2 (same sample as shown in Fig. 2b).

due to friction between the Si tip and the sample surface. Thus, the phase lag contrast between $NiPS_3$ and the SiO_2 substrate shows that the cantilever tip interacts differently with these two surfaces, demonstrating that significant chemical contrast is present.

Calibration of $NiPS_3$ thickness assignment

Due to the presence of a significant base height offset, it is important to clearly confirm that our layer number assignment is correct. We address this issue by studying partially overlapping thin sheets, similar to earlier studies on folded graphene sheets.[9] Figure S3a shows an AFM scan of 2-layer and 5-layer sheets that exhibit regions of overlap, along with height profiles across the sheets (Fig. S3b,c). AFM profiles that traverse the boundary between the SiO_2 substrate and the bilayer sheet are expected to feature a step height comprising two unit cells of $NiPS_3$ and the base height offset, $\Delta(N=2)+\Delta_{\text{offset}}$. In contrast, traces that traverse the boundary between 5-layer and (5+2)-layer areas will not be subject to an offset, and the resulting step height ought to simply correspond to two $NiPS_3$ unit cells, $\Delta(N=2)$. Table S2 lists the apparent step heights determined from the profiles shown in Fig. S3. The observed values $\Delta(N=2)$ are consistent with the expected thickness 1.27 nm of a $NiPS_3$ bilayer, providing an unambiguous confirmation of our thickness assignments. Moreover, inferred values of the base height offset are consistent with our previous determination shown in Fig. 2.

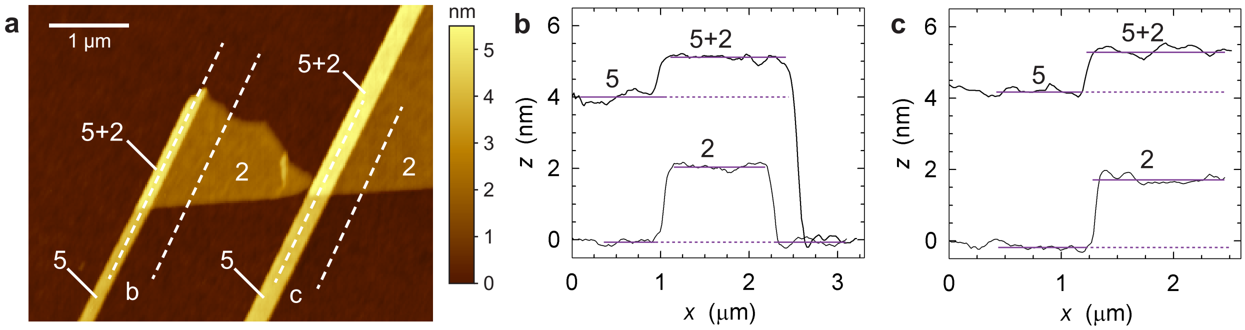

Supplementary Figure S3. Calibration of $NiPS_3$ sheet thicknesses measured by AFM. (a) Tapping-mode AFM topography image, comprising bilayer flakes (1) and pentalayer flakes (5), as well as areas of overlap (5+2). (b,c) Height profiles of along the lines indicated in (a).

	Apparent step height $\Delta(N=2)+\Delta_{\text{offset}}$ $=z(N=2)-z(SiO_2)$	Apparent step height $\Delta(N=2)$ $=z(N=5+2)-z(N=5)$	Base height offset Δ_{offset}
Trace (b)	2.14 nm	1.11 nm	1.03 nm
Trace (c)	1.89 nm	1.10 nm	0.79 nm

Supplementary Table S2. Apparent step heights between $NiPS_3$ sheets of different thickness, and between $NiPS_3$ sheets and the Si/SiO_2 substrate.

Effects of $NiPS_3$ sample aging

Exposure of exfoliated $NiPS_3$ to ambient results in gradual degradation of the sheets, leading to altered sample properties, as illustrated in Fig. S4. AFM measurements performed several months after sample exfoliation routinely yield $NiPS_3$ multilayer sheet heights that are ~ 1 nm greater than the heights determined directly after exfoliation (Fig. S4b,c). Changes in the optical properties of sheets due to degradation are readily observed after about a week. Optical brightfield photographs taken under identical lighting conditions immediately after exfoliation and 11 days later (Fig. S4d,e) show a contrast reduction that is particularly noticeable in 3- and 4-layer sheets. Furthermore, a comparison of the Raman spectra of pristine and degraded $NiPS_3$ trilayers shown in Fig. S4f reveals that spectral peaks corresponding to the $A_{1g}^{(2)}$ and $A_{1g}^{(3)}$ phonon modes are strongly attenuated in degraded trilayer sheets, and other peaks are suppressed entirely. Taken together, these observations suggest that sample degradation occurs by a gradual change of the chemical composition and crystal structure of exfoliated $NiPS_3$ sheets, though the precise degradation mechanism remains unclear at present.

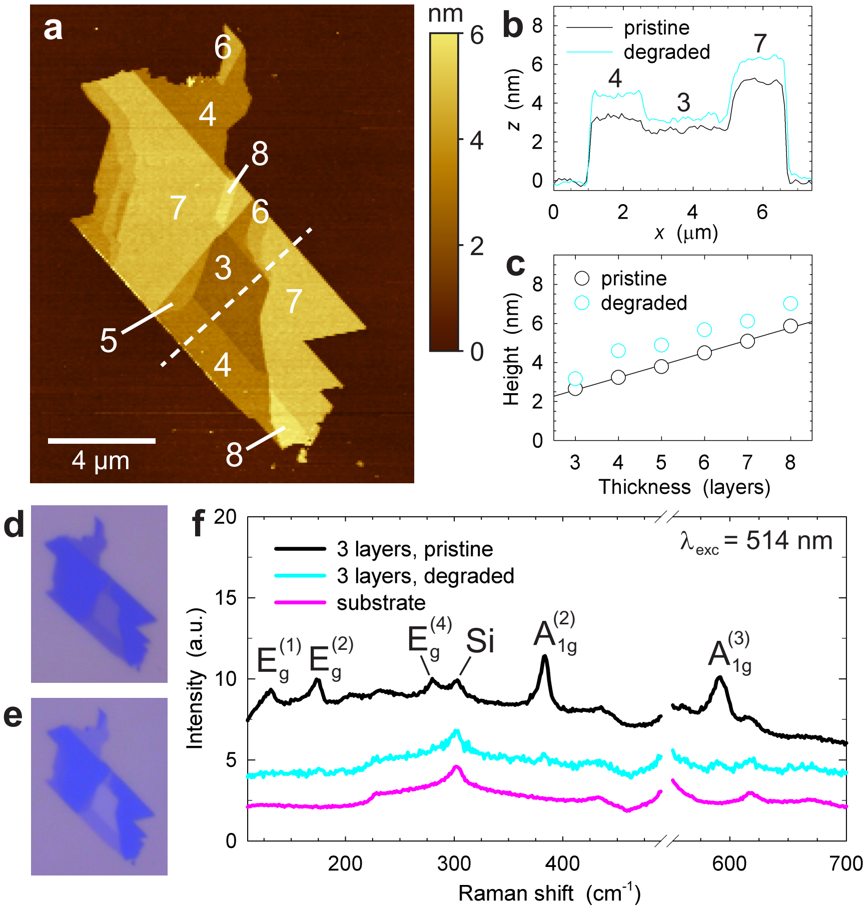

Supplementary Figure S4. Effects of $NiPS_3$ sample aging. (a) Tapping-mode AFM topography image of thin $NiPS_3$ sheets, $3-8$ layers indicated (same sample as shown in Fig. 2a). (b) Height profiles along the line shown in (a), directly after exfoliation (pristine) and several months later (degraded). (c) Apparent layer heights evaluated from AFM scans, for the pristine and degraded sample. (d,e) Brightfield microscope images of the same sample, (d) directly after exfoliation and (e) 11 days later. (f) Raman spectra of trilayer $NiPS_3$, acquired at $\lambda_{exc} = 514$ nm, for a pristine sheet and a degraded sample (11 days after exfoliation). For the degraded trilayer sheet, only remnants of the $A_{1g}^{(2)}$ and $A_{1g}^{(3)}$ modes remain. The spectrum of the degraded $NiPS_3$ sample was collected with an illumination power $\simeq 0.2$ mW, and its intensity has been scaled to match the spectrum of the pristine sample acquired at $\simeq 1$ mW. Data are offset vertically for clarity. The spectral region dominated by the Raman peak of the silicon substrate around 520 cm^{-1} has been omitted.

Supplementary Raman spectroscopic characterization

The eight phonon modes that are Raman-active in bulk MPS_3 are visualized in Fig. S5. The vibrational amplitudes and directions of all atoms contained in the $M_2P_2S_6$ unit cell are shown for the five doubly degenerate out-of-plane E_g phonon modes and the three in-plane A_{1g} phonon modes, based on the calculations of Bernasconi *et al.*[20] Table S3 lists the positions of the first-order Raman spectral features corresponding to these phonon modes in bulk $NiPS_3$, comparing theoretical calculations and experimental results of measurements performed at excitation wavelength 488 nm (ref. 20), and our results obtained at $\lambda_{\text{exc}} = 514$ and 633 nm.

Raman spectral data of thin $NiPS_3$ sheets shown in the main article are collected at $\lambda_{\text{exc}} = 633$ nm. Here we present analogous spectra for 514 nm excitation, acquired in the same session and on the same sample. Relevant observations from these spectra are discussed in the main text. Figure S6 shows the Raman response of exfoliated thin $NiPS_3$ sheets, for $2-7$ layers thickness, as well as for thick sheets and the substrate. The spectral regions corresponding to the A_{1g} out-of-plane phonon modes are shown in detail in Fig. S7. Spectral data for phonon modes $A_{1g}^{(2)}$ and $A_{1g}^{(3)}$ are decomposed into Lorentzian line shapes by a least-squares fit procedure. Among the out-of-plane phonon modes, spectral data of the $E_g^{(2)}$ have a favorably high signal-to-noise ratio, and Fig. S8 presents the Raman spectra of this mode, measured at $\lambda_{\text{exc}} = 514$ and 633 nm. For excitation at 633 nm, spectra can be decomposed into Lorentzian line shapes, as discussed in the main text.

Figure S9a shows the integrated intensity ratios $I(514\ \text{nm})/I(633\ \text{nm})$ for the main peaks of phonon modes $A_{1g}^{(2)}$, $A_{1g}^{(3)}$, and $E_g^{(2)}$, all of which have a strong Raman response both for 514 and 633 nm excitation. The intensity ratios of the $E_g^{(2)}$, $A_{1g}^{(1)}$, and $A_{1g}^{(3)}$ modes with respect to the $A_{1g}^{(2)}$ mode are shown in Fig. S9b,c,d for $\lambda_{\text{exc}} = 514$ and 633 nm.

The second-order Raman response that appears in the spectral regions around 800 and 1180 cm^{-1} depends significantly on the excitation wavelength. Figure S10 shows the spectra corresponding to $2A_{1g}^{(2)}$ and $2A_{1g}^{(3)}$ processes for 514 nm excitation.

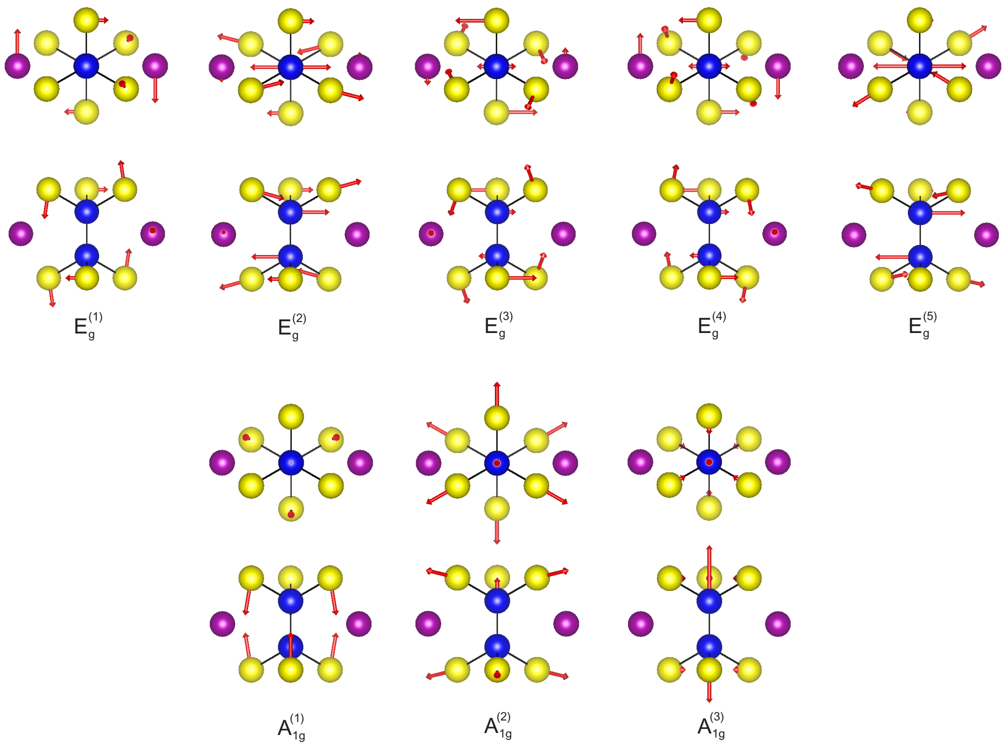

Supplementary Figure S5. Schematic representation (top view, side view) of vibrational amplitudes of $M_2P_2S_6$ unit cell atoms, for the five in-plane E_g and three out-of-plane A_{1g} phonon modes.[20] Covalent bonds within $(P_2S_6)^{4-}$ anions (black lines) are indicated.

	Theoretical (ref. 20)	Experimental $\lambda_{exc} = 488$ nm (ref. 20)	This work $\lambda_{exc} = 514$ nm	This work $\lambda_{exc} = 633$ nm
$E_g^{(1)}$	122	131	130	131
$E_g^{(2)}$	160	176	175	176.1
$E_g^{(3)}$	204	236	absent	234
$A_{1g}^{(1)}$	250	253	absent	253.9
$E_g^{(4)}$	280	280	277	280
$A_{1g}^{(2)}$	395	384	381.3	383.5
$E_g^{(5)}$	559	560	557	$\sim$ 560 (very weak)
$A_{1g}^{(3)}$	594	588	587.0	587.6

Supplementary Table S3. Raman spectral peak positions (in cm^{-1}) for all Raman-active first-order phonon modes of bulk $NiPS_3$. Calculated and measured values reported in previous work,[20] and peak positions experimentally determined in this work. We provide wavenumber values to four significant digits whenever fits of Lorentzian line shapes to spectral data allow for a more precise determination, and to three significant digits otherwise.

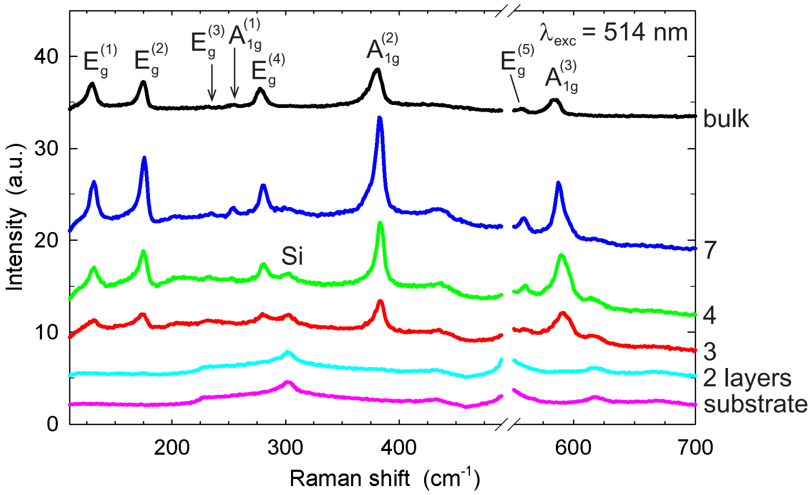

Supplementary Figure S6. Raman spectra of thin $NiPS_3$ sheets ($2-7$ layers), measured at $\lambda_{exc} = 514$ nm, together with the spectrum of a thick sheet ($d \approx 107$ nm), and the substrate spectrum. The positions of 5 in-plane E_g and 3 out-of-plane A_{1g} phonon modes are indicated. All spectra were acquired in a single pass, under identical experimental conditions, on the sample shown in Fig. 1d. Spectra have not been scaled; data are offset vertically for clarity. The spectral region dominated by the Raman peak of the silicon substrate around 520 cm^{-1} has been omitted.

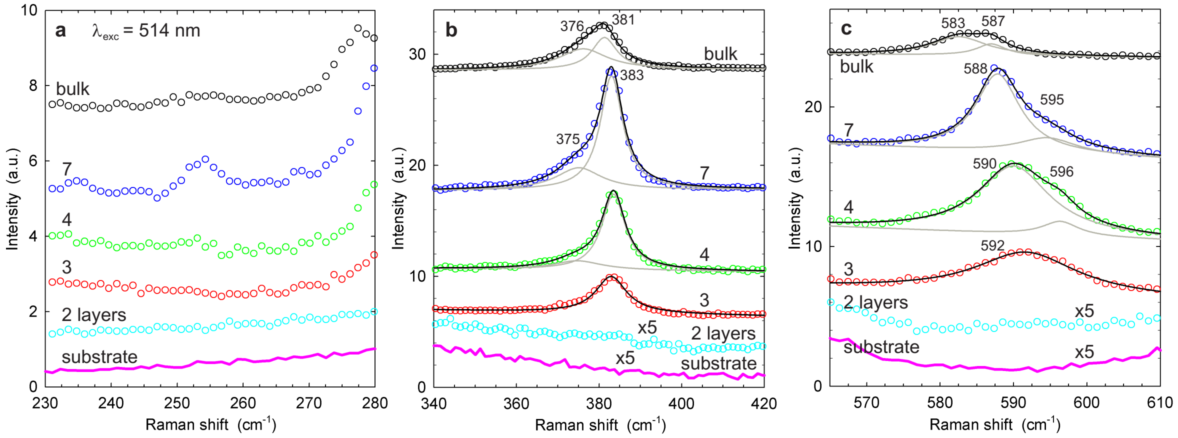

Supplementary Figure S7. Raman spectral features of out-of-plane A_{1g} phonon modes, for excitation at 514 nm. (a) $A_{1g}^{(1)}$ mode. This mode is extremely weak at $\lambda_{exc} = 514$ nm. (b) $A_{1g}^{(2)}$ mode, (c) $A_{1g}^{(3)}$ mode. Raman spectra of 4-layer, 7-layer and thick $NiPS_3$ sheets are composed of two distinct Lorentzian lines: individual Lorentzian fit curves (grey lines), and sum of fit curves (black lines). Data of bilayer sample and substrate have been magnified by factor 5 where indicated.

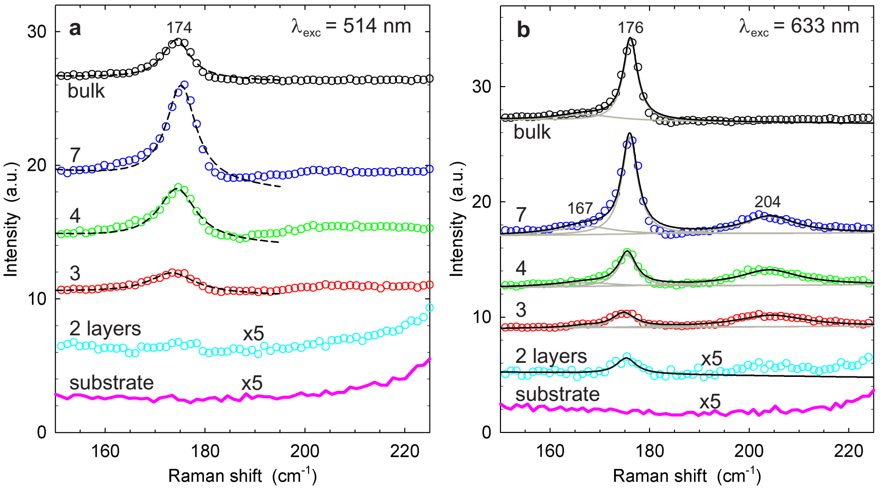

Supplementary Figure S8. Raman spectral features of doubly degenerate in-plane $E_g^{(2)}$ phonon mode, at excitation wavelengths (a) 514 nm, (b) 633 nm. For case (b), data can be fit with three Lorentzian peaks: individual Lorentzian fit curves (grey lines), and sum of fit curves (black lines). Labels state wavelength shifts (in cm^{-1}) of individual peak centers. For case (a), fits involving a single Lorentzian peak (dashed lines) are shown. The low intensity of secondary peaks at $\simeq 167$ and 204 cm^{-1} precludes robust fitting of spectra. Data of bilayer sample and substrate have been magnified by factor 5. Spectra are offset vertically for clarity.

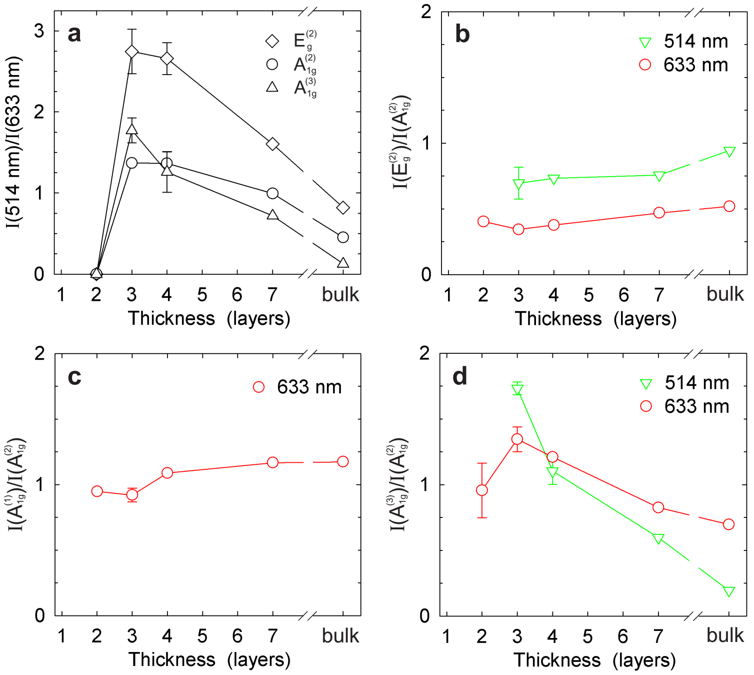

Supplementary Figure S9. Integrated intensity ratios of dominant Raman-active phonon modes. (a) Ratio of Raman response intensity at 514 nm *versus* 633 nm excitation. (b) Ratio of $E_g^{(2)}$ mode intensity, with respect to the $A_{1g}^{(2)}$ mode intensity. (c) Analogous information for $A_{1g}^{(1)}$ mode, (d) for $A_{1g}^{(3)}$ mode.

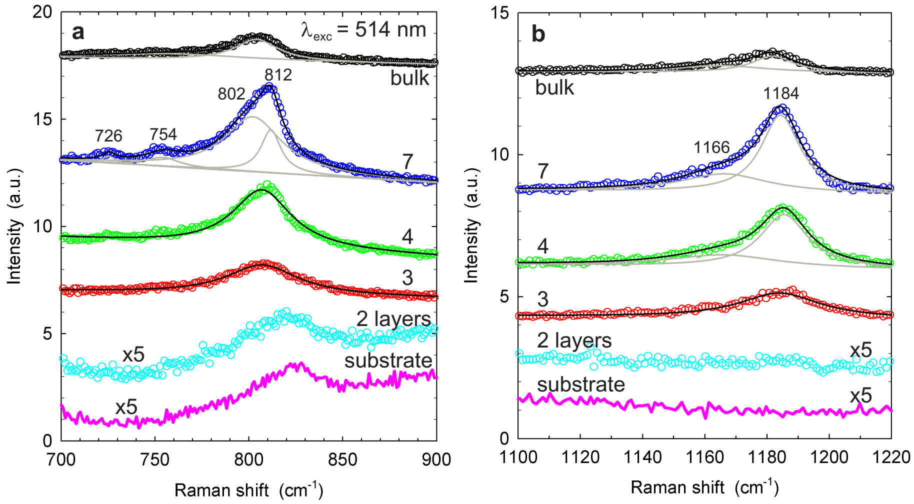

Supplementary Figure S10. Raman spectral features of second order processes, for excitation at 514 nm. Spectral range containing (a) $2A_{1g}^{(2)}$ second-order processes, (b) $2A_{1g}^{(3)}$ second-order processes. Spectra are described by superpositions of up to 4 Lorentzian peak shapes. Individual Lorentzian fit curves, grey lines. Sum of fit curves, black lines. Labels state wavelength shifts (in cm^{-1}) of individual peak centers. Data of bilayer sample and substrate have been magnified by factor 5. Spectra are offset vertically for clarity.

References

1. Brec, R. Review on Structural and Chemical Properties of Transition Metal Phosphorous Trisulfides MPS_3. *Solid State Ionics* **22**, 3–30 (1986).
2. Brec, R., Schleich, D. M., Ouvrard, G., Louisy, A. & Rouxel, J. Physical Properties of Lithium Intercalation Compounds of the Layered Transition Chalcogenophosphates. *Inorg. Chem.* **18**, 1814–1818 (1979).
3. Le Flem, G., Brec, R., Ouvard, G., Louisy, A. & Segransan, P. Magnetic Interactions in the Layer Compounds MPX_3 (M = Mn, Fe, Ni; X = S, Se). *J. Phys. Chem. Solids* **43**, 455–461 (1982).
4. Kurosawa, K., Saito, S. & Yamaguchi, Y. Neutron Diffraction Study on $MnPS_3$ and $FePS_3$. *J. Phys. Soc. Japan* **52**, 3919–3926 (1983).
5. Ouvrard, G., Brec, R. & Rouxel, J. Structural Determination of Some MPS_3 Layered Phases (M = Mn, Fe, Co, Ni and Cd). *Mater. Res. Bull.* **20**, 1181–1189 (1985).
6. Prouzet, E., Ouvrard, G. & Brec, R. Structure Determination of $ZnPS_3$. *Mater. Res. Bull.* **21**, 195–200 (1986).
7. Taylor, B. E., Steger, J. & Wold, A. Preparation and Properties of Some Transition Metal Phosphorus Trisulfide Compounds. *J. Solid State Chem.* **7**, 461–467 (1973).
8. Joy, P. A. & Vasudevan, S. Magnetism in the Layered Transition-Metal Thiophosphates MPS_3 (M = Mn, Fe, and Ni). *Phys. Rev. B* **46**, 5425–5433 (1992).
9. Novoselov, K. S. *et al.* Electric Field Effect in Atomically Thin Carbon Films. *Science* **306**, 666–669 (2004).
10. Gupta, A., Chen, G., Joshi, P., Tadigadapa, S. & Eklund, P. C. Raman Scattering from High-Frequency Phonons in Supported n-Graphene Layer Films. *Nano Lett.* **6**, 2667–2673 (2006).
11. Li, S.-L. *et al.* Quantitative Raman Spectrum and Reliable Thickness Identification for Atomic Layers on Insulating Substrates. *ACS Nano* **6**, 7381–7388 (2012).
12. Chakraborty, B., Matte, H. S. S. R., Sood, A. K. & Rao, C. N. R. Layer-Dependent Resonant Raman Scattering of a Few Layer MoS_2. *J. Raman Spectrosc.* **44**, 92–96 (2013).
13. Li, H. *et al.* From Bulk to Monolayer MoS_2: Evolution of Raman Scattering. *Adv. Funct. Mater.* **22**, 1385–1390 (2012).
14. Yamamoto, M. *et al.* Strong Enhancement of Raman Scattering from a Bulk-Inactive Vibrational Mode in Few-Layer $MoTe_2$. *ACS Nano* **8**, 3895–3903 (2014).
15. Hajiyev, P., Cong, C., Qiu, C. & Yu, T. Contrast and Raman Spectroscopy Study of Single- and Few-Layered Charge Density Wave Material: 2H-$TaSe_2$. *Sci. Rep.* **3**, 2593 (2013).
16. Chen, L., Yu, X. & Wang, D. Cantilever Dynamics and Quality Factor Control in AC Mode AFM Height Measurements. *Ultramicroscopy* **107**, 275–280 (2007).
17. Nemes-Incze, P., Osváth, Z., Kamarás, K. & Biró, L. Anomalies in Thickness Measurements of Graphene and Few Layer Graphite Crystals by Tapping Mode Atomic Force Microscopy. *Carbon* **46**, 1435–1442 (2008).
18. Ishigami, M., Chen, J., Cullen, W., Fuhrer, M. & Williams, E. Atomic Structure of Graphene on SiO_2. *Nano Lett.* **7**, 1643–1648 (2007).
19. Xu, K., Cao, P. & Heath, J. Graphene Visualizes the First Water Adlayers on Mica at Ambient Conditions. *Science* **329**, 1188–1191 (2010).
20. Bernasconi, M. *et al.* Lattice Dynamics of Layered MPX_3 (M = Mn, Fe, Ni, Zn; X = S, Se) Compounds. *Phys. Rev. B* **38**, 12089–12099 (1988).
